# Chest Compressions as a Precipitant of Aortocaval Hematoma and Pseudoaneurysm in the Setting of Inferior Vena Cava (IVC) Filter Aortic Penetration: A Case Report

**DOI:** 10.7759/cureus.108966

**Published:** 2026-05-16

**Authors:** Anthony N Cholagh, Deema Ujayli, Ragamayi Maramraju, Mansi Joglekar, Rohan Patel

**Affiliations:** 1 Internal Medicine, Henry Ford Hospital, Detroit, USA; 2 Internal Medicine, Henry Ford Health System, Detroit, USA

**Keywords:** aorta injury, aortic injury, ct (computed tomography) imaging, endovascular aortic repair (evar), ivc filter complication

## Abstract

Inferior vena cava (IVC) filters are associated with delayed complications, including aortic penetration, which may present asymptomatically or with clinical sequelae warranting vascular intervention. We present a case of a 52-year-old male with a known indwelling IVC filter with aortic penetration who developed an aortocaval hematoma and pseudoaneurysm following a brief resuscitative event involving chest compressions. This case was managed successfully with endovascular aortic repair (EVAR), with no residual pseudoaneurysm or active extravasation on post-procedural imaging. This case underscores the importance of timely IVC filter retrieval when clinically appropriate and the need for active long-term surveillance in patients with indwelling retrievable IVC filters to mitigate the risk of delayed and potentially life-threatening complications.

## Introduction

Inferior vena cava (IVC) filters are mechanical devices placed within the infrarenal IVC to prevent pulmonary embolism (PE) in patients with deep vein thrombosis (DVT) who have an absolute contraindication to anticoagulation, have experienced prior failure of therapeutic anticoagulation, or have developed anticoagulation-related complications [1,2]. These devices are broadly categorized as either permanent or retrievable, with retrievable filters designed for removal once the underlying thromboembolic risk has resolved. The SAFE-IVC study, a retrospective observational study, demonstrated that IVC filter retrieval rates remain exceptionally low, with a cumulative retrieval incidence of only 15.3% at a median follow-up of 1.2 years and 16.8% at a maximum follow-up of 9.0 years [3]. This is concerning, as the likelihood of successful filter retrieval decreases over time and is associated with higher rates of filter-related complications [3,4].

Delayed IVC filter-related complications include migration, angulation, fracture, caval thrombosis, iliac thrombosis, filter thrombus, fragment embolization, and IVC penetration [5]. A systematic literature review of 9,002 patients identified IVC penetration in 19% of subjects, with 19% of those patients demonstrating evidence of adjacent organ or structure involvement [6]. Aortic penetration is rare, with a systematic review identifying only 53 cases reported between January 1967 and April 2025 [7].

We present a case of an indwelling IVC filter with strut penetration into the abdominal aortic lumen, complicated by a new aortocaval hematoma and pseudoaneurysm following a brief resuscitative event, which underscores the diagnostic and management challenges associated with this rare and potentially life-threatening complication.

## Case presentation

**Figure 1:**
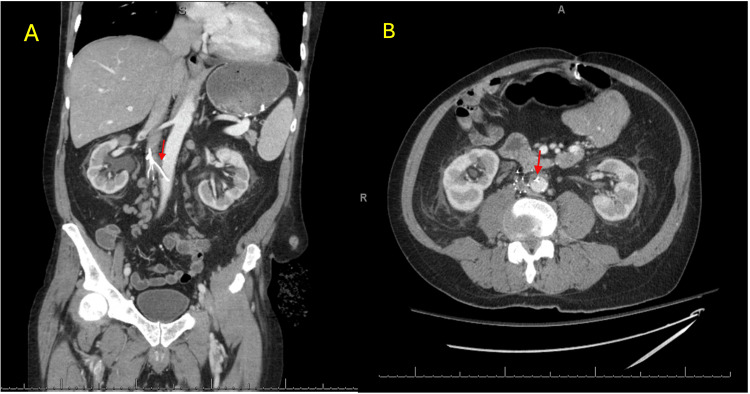
Computed tomography (CT) scan demonstrating IVC filter penetration into the aortic lumen A) Coronal view with the red arrows demonstrating the site of aortic penetration with no extravasation. B) Axial view with the red arrows demonstrating the site of aortic penetration with no extravasation.

A 52-year-old male with a past medical history of Crohn's disease status post left colectomy, chronic bilateral lower extremity deep vein thrombosis (DVT) status post IVC filter placement (2008), and multiple myeloma presented with abdominal pain and increased ostomy output. Gastroenterology was consulted for concern for a Crohn's flare in the setting of an elevated C-reactive protein (CRP) on admission, which was 4.6 mg/dL (normal range is <0.5 mg/dL). The patient was initiated on intravenous (IV) corticosteroids with initial improvement in symptoms. However, his pain recurred after transition to oral corticosteroids, prompting gastroenterology to recommend CT enterography (CTE) to evaluate for an abscess or infection. CTE demonstrated no evidence of abscess or infection but revealed terminal ileitis and an indwelling IVC filter with one tine extending into the aortic lumen (Figure 1). This finding had been previously documented and remained stable without associated complications.

**Figure 2:**
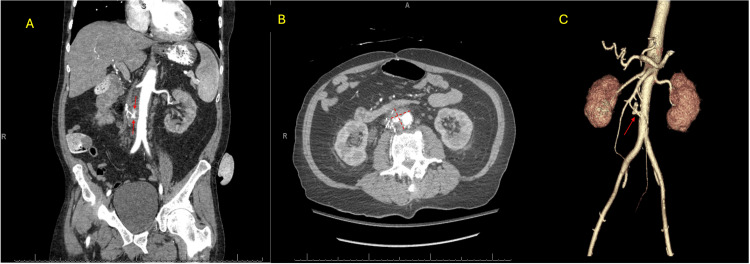
CT scan with 3D reconstruction demonstrating active extravasation A) Coronal view demonstrating active extravasation noted by the bright contrast (red arrows) B) Axial view that demonstrates the aortocaval hematoma (red arrows) that measures 3.2cm x 2cm. C) 3D reconstruction of IVC filter penetrating the aorta (red arrow).

The hospital course was subsequently complicated by a brief episode of unresponsiveness, during which ancillary staff administered chest compressions prior to spontaneous recovery of consciousness. The patient was found to be hypotensive and was managed with IV fluid resuscitation. A broad infectious workup was initiated, which identified methicillin-resistant *Staphylococcus aureus* (MRSA) bacteremia, suspected to be secondary to his indwelling implanted port. Repeat CT of the abdomen and pelvis, obtained as part of the workup, demonstrated active contrast extravasation from the aorta with an associated aortocaval hematoma (Figure 2).

**Figure 3:**
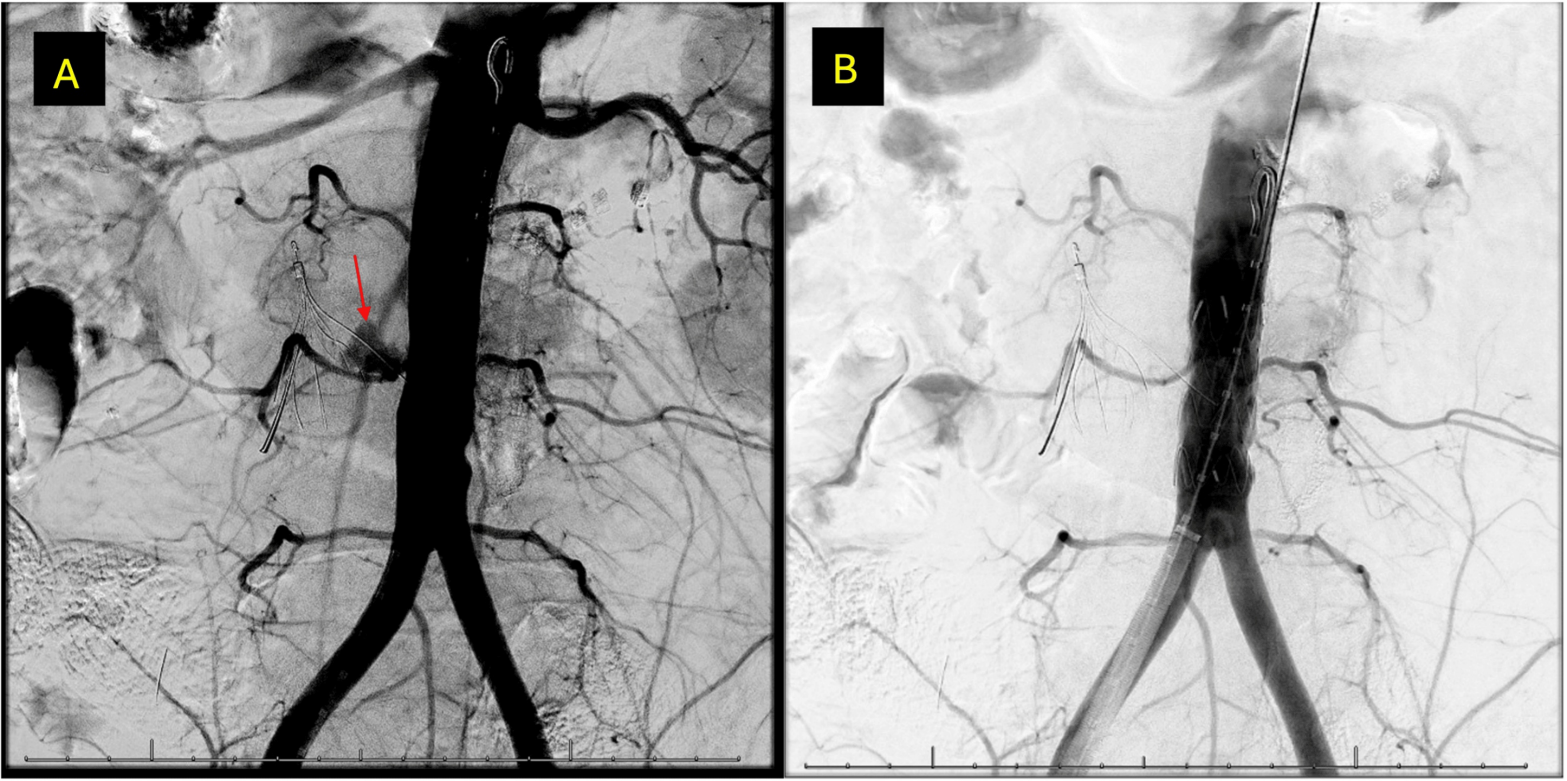
Intraoperative angiography of the aorta A) Pseudoaneurysm at the site of the strut penetration (red arrow). B) Resolution of the pseudoaneurysm after the Gore graft extender was placed.

**Figure 4:**
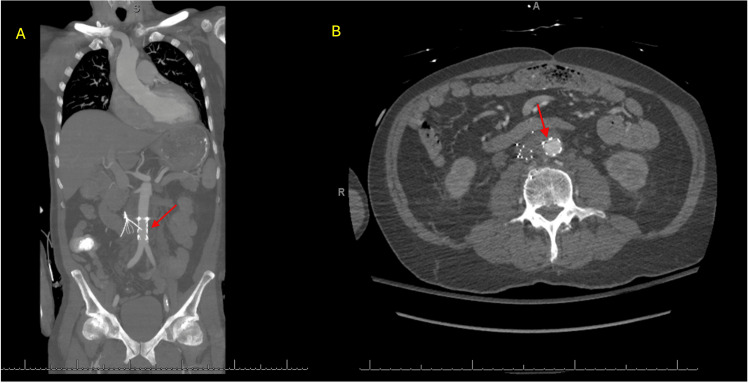
CT demonstrating endovascular aortic repair (EVAR) with resolution of active extravasation A) Coronal view demonstrating the Gore extender graft (red arrow) with resolution of active extravasation. B) Axial view demonstrating the Gore extender graft (red arrow) with resolution of active extravasation.

Vascular surgery was consulted, and the patient was taken to the operating room for endovascular aortic repair (EVAR). Intraoperative angiography demonstrated a pseudoaneurysm at the site of IVC filter strut penetration. A Gore aortic extender graft was deployed, with successful exclusion of the pseudoaneurysm confirmed on post-surgical angiography (Figure 3). Repeat CT of the abdomen and pelvis obtained the following day, demonstrated no residual pseudoaneurysm or active extravasation (Figure 4).

## Discussion

Aortic penetration by an IVC filter is an exceedingly rare but well-documented complication. The precise mechanism by which penetration occurs remains incompletely understood; however, multiple contributing factors have been proposed. Current data suggest that conically shaped filters with free struts have a higher incidence of IVC penetration compared to those without free struts [8]. Additional factors, including the outward radial force exerted by the filter, the sharpness of the filter barbs, and the mechanical integrity of the native IVC wall, have been proposed as predictors of penetration risk [8,9]. Elevated intra-abdominal pressure secondary to prolonged coughing or obesity may also contribute by displacing the filter toward the aorta [6].

Several cases of new or worsened IVC filter penetration following a traumatic precipitant have been reported, including patients involved in motor vehicle accidents, those who underwent recent abdominal surgery, and individuals engaging in strenuous exercise [10-12]. The present case is notable in that, to our knowledge, no prior cases of aortocaval hematoma and pseudoaneurysm following chest compressions in a patient with a known IVC filter aortic penetration have been reported in the literature.

Management remains largely individualized in the absence of formal guidelines. Among the 53 reported cases of IVC filter penetration into the aorta, 34 patients underwent IVC filter retrieval via endovascular or surgical approach, nine underwent aortic repair without filter retrieval, six were managed conservatively, one died prior to intervention, and one declined intervention [7].

In the present case, the vascular surgery team determined that IVC filter removal carried a significant risk for significant vascular injury; therefore, EVAR was pursued with the filter left in situ.

In the future, active surveillance protocols for patients with indwelling retrievable IVC filters should be prioritized, as prolonged filter dwell time is associated with an increased risk of complications and a decreased likelihood of successful retrieval.

## Conclusions

IVC filter placement is indicated in patients with known DVT who have contraindications to anticoagulation for the prevention of PE. Complications of indwelling IVC filters, including aortic penetration, become more likely with prolonged filter dwell time, a concern that is compounded by the consistently low rates of filter retrieval observed in the literature. This case highlights a rare and previously unreported complication, aortocaval hematoma and pseudoaneurysm following chest compressions in the setting of known IVC filter aortic penetration, and underscores the critical importance of timely filter retrieval and active long-term surveillance in patients with indwelling retrievable IVC filters.
